# Using Probiotics to Mute *Salmonella enteric* Serovar Typhimurium: An Opinion

**DOI:** 10.3389/fbioe.2020.00558

**Published:** 2020-06-23

**Authors:** Yang Shi, Juan Li, Yihao Shen, Zhongke Sun

**Affiliations:** ^1^Institute of Food and Drug Inspection, Zhoukou Normal University, Zhoukou, China; ^2^College of Chemistry and Molecular Engineering, Zhengzhou University, Zhengzhou, China

**Keywords:** *Salmonella enteric* serovar Typhimurium, probiotic intervention, genetically modified probiotics, probiotic formulation, synthetic biology

## Introduction

**Figure 1:**
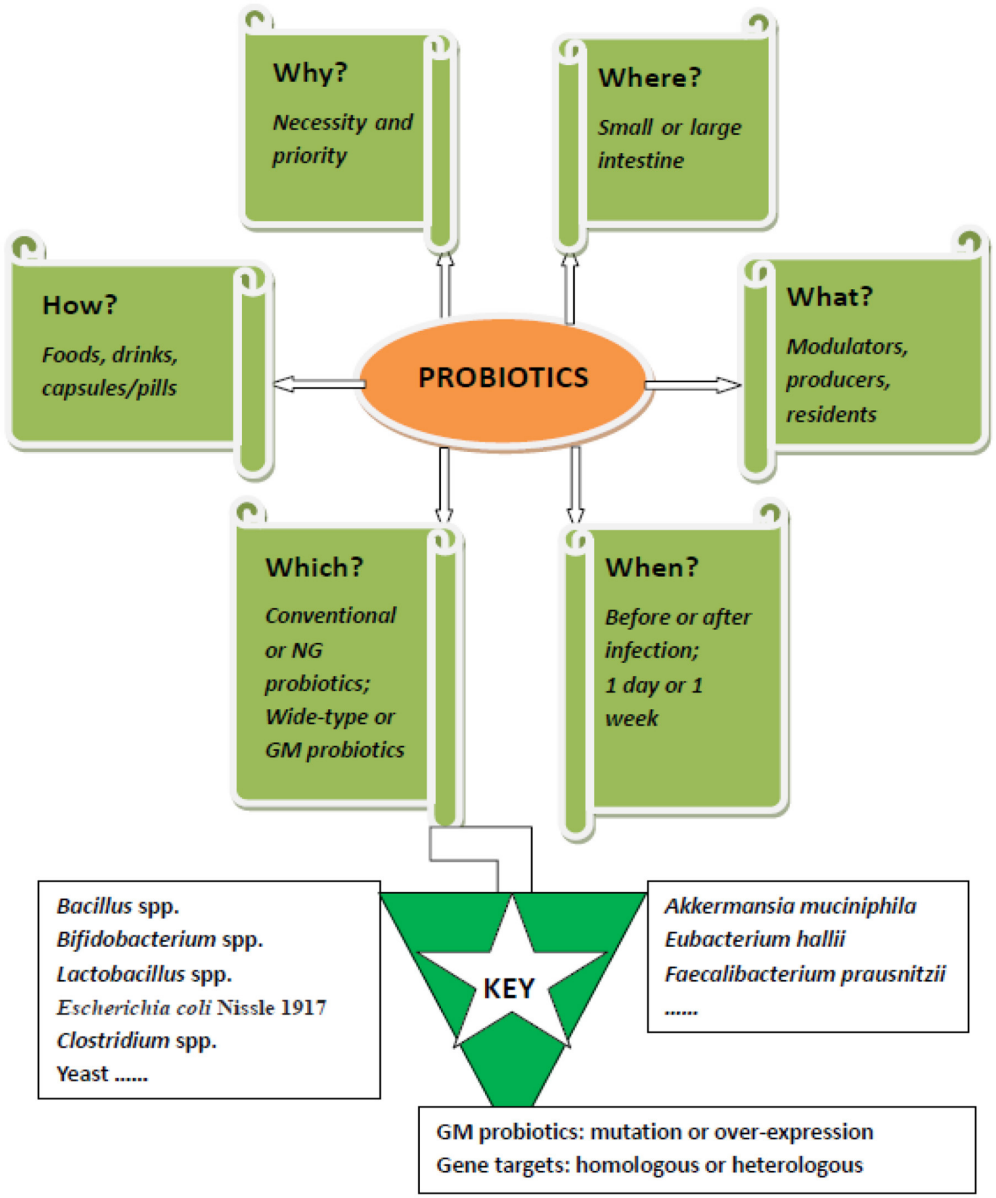
The 5W1H framework of probiotics muting *Salmonella enteric* serovar Typhimurium: For effective intervention of salmonella infection by probiotics, six questions need to be answered. 1. Why is probiotic intervention necessary? 2. Where do probiotics exert their anti-salmonella effects? 3. What roles do probiotics play during treatment? 4. When should probiotics be used? 5. Which probiotic strain should be used for either prophylactic or therapeutic treatment? 6. How should probiotics be formulated for oral delivery? NG, next generation; GM, genetically modified.

A recent study reported on the prevention of enteric bacterial infections, such as pathogenic *Salmonella enteric* serovar Typhimurium (ST), by oral consumption of a genetically modified (GM) probiotic strain in mice (Peng et al., 2019). The study highlighted the effect of GM probiotics by overproducing conjugated linoleic acids (CLA) in *Lactobacillus casei* (*L. casei*), and a significantly improved preventive effect was displayed. However, successful probiotic intervention of ST, one of the most prevalent foodborne pathogens, is challenging, and more work is necessary. We know that many probiotic strains have an inhibitive effect on ST by different mechanisms (Adetoye et al., 2018; Pradhan et al., 2019). In addition, several factors restrain the effectiveness of potential probiotic therapy, such as intervention time, routine, and formulation (Sun et al., 2020). To provide a brief but more complete view of ST prevention or even elimination by using probiotics, this opinion is presented. The 5W1H questions (why, where, what, when, which, and how) on this important topic are answered and broadly discussed (Figure 1), aiming to facilitate a comprehensive understanding of this promising therapy.

## Why Use Probiotics?

ST is a Gram-negative, non-spore-forming, facultative anaerobic bacterium and can infect any warm-blooded animal (Gut et al., 2018). The bacterium is mainly transmitted through fecal–oral routes, and susceptible hosts get ST through contaminated foods and water. Currently, ST is responsible for millions of infections worldwide and presents a cosmopolitan distribution in animal-based food matrices (Ferrari et al., 2019). People infected with ST normally develop diarrhea, fever, and abdominal cramps. In some severe cases, ST may spread from the intestines to the bloodstream and then to other body sites, causing death (https://www.cdc.gov/salmonella/).

At present, ST is mainly treated with antibiotics, including ciprofloxacin, ceftriaxone, and ampicillin. However, multiple drug–resistant (MDR) ST is rapidly expanding (Obaidat and Stringer, 2019). This antibiotic resistance has led to failed treatment of ST in clinics and resulted in high mortality and morbidity. Overuse of antibiotics is also associated with gut dysbiosis and induces other disorders, such as inflammatory bowel disease or allergies (Schulfer et al., 2018). Therefore, probiotics have been identified as a promising solution, in both the preventive and therapeutic treatment of ST. Probiotics are generally recognized as safe (GRAS), possessing many benefits for humans and animals, such as protective effects against pathogenic infection and modulation of gut microbiota (Hill et al., 2014).

## Where Do Probiotics Work?

In chickens, ST first attaches to the cecal epithelial cells and then spreads to the liver, spleen, and oviduct. In pigs, early ST infection disrupts microbiome composition and functionality principally at the ileum (Argüello et al., 2018). In humans, ST robustly colonizes the distal parts of the intestine, such as the cecum and colon (Lam and Monack, 2014). Therefore, probiotic strains may work at different sites (jejunum, ileum, colon, and cecum) in different hosts. This is an important factor affecting their efficacies. It has been revealed that cecal colonization is critical to ST transmission along the food chain. Reducing ST colonization in the cecum could be the front line for probiotic intervention in humans. As has been shown, GM *L. casei* promotes overall bacterial species diversity and increases the abundance of *Lactobacillus* and *Bifidobacterium* in the cecum in mice (Peng et al., 2019). However, gut microbes, including probiotics, have their biogeography [e.g., *Lactobacillus* spp. is mainly in the small intestine, and *Bifidobacterium* spp. is mainly in the large intestine (Donaldson et al., 2016)].

## What Roles Do Probiotics Play?

Probiotics play important roles in human health. As normal commensals, they exert their prophylactic and therapeutic properties against ST in four main ways (Gut et al., 2018). First, they protect the tight junction in the gut and modulate both innate and acquired immunity of the host (Pedicord et al., 2016; Thiemann et al., 2017). Second, they directly compete with ST for niches and nutrients, like binding sites and iron (Deriu et al., 2013; Lam and Monack, 2014). Third, they produce various harmful substances to ST, such as antimicrobial molecules (Kanmani et al., 2013; Garcia-Gutierrez et al., 2019). Fourth, they modulate the virulence of ST by regulating the expression of corresponding genes (Tanner et al., 2016).

In fact, probiotics can be modulators, producers, and residents after being administered. As modulators, probiotics effectively modulate either the host or the pathogen. *L. casei* modulates host immunity by regulating the expression of intestinal inflammation-related cytokines [e.g., suppressing pro-inflammatory cytokines and provoking anti-inflammatory cytokines after ST infection (Peng et al., 2019)]. Several *Lactobacillus* spp. modulate ST by regulating gene expression related to colonization and virulence (Muyyarikkandy and Amalaradjou, 2017). In addition, probiotics modulate gut microbiota homeostasis and change the microbial composition, which is regarded as the intersection or front line for curing many infectious diseases (Cani, 2017). As producers, probiotics produce metabolites that may be signals to stimulate host immunity or substances to inhibit ST colonization and growth. For example, *L. pentosus* AT6 and its cell-free culture supernatants inhibit ST growth and its adhesion as well as invasion (Liu et al., 2018). As residents, probiotics themselves are able to reduce ST through physical repellence and colonization resistance (Ubeda et al., 2017). By sharing the same habitant, they also compete with ST for limited nutrients (Deriu et al., 2013).

## When To Use Probiotics?

Pre-administration of probiotics is an effective method demonstrated in animal studies. For example, 1-week pre-administration of either wild-type *L. casei* or the GM counterpart has displayed a significant protective effect on ST infection (Peng et al., 2019). It has been reported that feeding probiotics 24 h before *Salmonella enteritidis* infection is efficacious in broilers, but data on prophylactic treatment timing regarding ST is not available (Higgins et al., 2010). Even though prevention from infection is somewhat more economically important than therapy after infection, strict prevention is often impossible as many causes are out of control. Therefore, probiotics have also been evaluated for their therapeutic effects after infection. Usually, salmonellosis begins to demonstrate symptoms 6 h to 4 days after ST infection and lasts 4–7 days. We need to know when to start intervention and how long probiotics should be used for full elimination of ST. Practically, we have to know whether prevention of ST really needs 1 week of pre-administration and whether 1–3 days administration after infection is diagnosed is enough to efficiently alleviate symptoms.

## Which Probiotics Should Be Used?

Probiotics are any non-pathogenic microorganisms that confer health-promoting properties when administered in adequate amounts (Hill et al., 2014). Therefore, probiotics can be either prokaryotes or eukaryotes, consisting of species belonging to *Bacillus* spp., *Bifidobacterium* spp., *Clostridium* spp., *Escherichia coli* Nissle 1917, and *Lactobacillus* spp., yeast, and so on (Kanmani et al., 2013). Currently, both conventional and next-generation probiotics are widely studied. Conventional probiotics, such as *Bifidobacterium* spp. and *Lactobacillus* spp., harbor most of the well-characterized probiotic strains and are widely commercialized. These probiotics reduce more than 90% of caecal ST load, prevent invasion of organs, and even completely eradicate ST (Gut et al., 2018). However, among so many probiotic strains, it is yet unknown which species/strains should be prioritized. In contrast to conventional probiotics, several next-generation (NG) probiotics have recently been identified, such as *Akkermansia muciniphila, Eubacterium hallii*, and *Faecalibacterium prausnitzii* (Almeida et al., 2019). The potential of these strains in muting ST is worth further investigation.

Besides the traditional application of wild-type probiotics, GM probiotics are also studied. Although there are safety issues, GM probiotics attracted much interest due to their extra advantages and strengthened effects (Barra et al., 2020). Two different approaches are used to construct advanced GM probiotics: mutation and overexpression. After genetic engineering, probiotic strains may strengthen their fitness in the gut and produce inhibitive substances much more. Considering the infection stages of ST, probiotic mutants with high adaptability may be more suitable for prevention, and specifically targeted overproduced probiotics may be more effective for therapy. For example, expression of microcin H47 in probiotic *E. coli* inhibits ST growth with improvement in fitness (Palmer et al., 2018). Overexpression of myosin cross-reactive antigen gene in *L. casei* improves the protective effect on ST more than the wild-type strain by increasing CLA (Peng et al., 2019). Other proteins and metabolites, such as bacterial peptidoglycan hydrolase (SagA), different kinds of antimicrobials, and even propionate, have an inhibitive effect on ST, guaranteeing the potential of GM probiotics, producing them for mutation of ST (Rangan et al., 2016; Sassone-Corsi et al., 2016; Jacobson et al., 2018). Based on the mechanisms of ST infection and prevention, versatile genes can be delivered by using probiotics as hosts in the future. Therefore, determining genes targeting different processes of ST infection may be equally as important as choosing a proper probiotic host. In our opinion, compared to conventional and NG probiotics, GM probiotics may be the best option for ST intervention albeit having inadequate investigation, particularly considering their strengthened and newly added effects.

Along with the rapid development of synthetic biology, interest has increased on the design and construction of GM probiotics as live biotherapeutics for a range of medical applications (Chua et al., 2017; Mays and Nair, 2018; Aggarwal et al., 2020). Synthetic biology explores diverse biosynthetic pathways and provides versatile engineering toolboxes for probiotic strain improvement (Yadav and Shukla, 2020). These toolboxes, including genetic circuits, different delivery systems, and a large number of genome-editing tools, successfully accelerated the development of advanced GM probiotics (Bober et al., 2018; Ozdemir et al., 2018). Live therapeutic GM probiotics can be constructed to function potentially as biosensors, regulators, delivery devices, and others for fighting against ST (Pedrolli et al., 2019; Barra et al., 2020). As applied for sensing and killing *Pseudomonas aeruginosa* and reducing vancomycin-resistant *Enterococcus* by GM probiotic *E. coli* Nissle 1917, similar approaches might be also promising for ST prevention (Hwang et al., 2017; Geldart et al., 2018). Moreover, a newly developed strategy called inducible plasmid self-destruction (IPSD) provides a novel genome-editing tool for simple gene knockout and knock-in in lactobacilli and bifidobacteria (Zuo et al., 2019). All these advances will strengthen both the prophylactic and therapeutic activity of GM probiotics on ST.

## How Should Probiotics Be Formulated?

As pointed out, formulation of viable probiotics while enabling cost-effective biomass yield is a critical step toward product development of translational application (Almeida et al., 2019). Regarding ST prevention, conventional probiotics (wild type) formulated as foods or drinks containing 10^6^ CFU/g or CFU/mL viable cells may be an acceptable way. Considering the GRAS status, consumption of them can be without control. However, to cure ST infection, NG or GM probiotics are more promising. These probiotics can be formulated as concentrated pills or capsules containing more than 10^9^ CFU/g or even more cells. They may be more suitable as over-the-counter drugs. Nevertheless, for any purposes, release of both conventional and GM probiotics as live biotherapeutical products in the market needs full assessment of safety (O'Toole et al., 2017).

## Author Contributions

ZS conceived the opinion. YS and JL wrote the draft manuscript. YS collected reference and drawn the figure. ZS finalized the manuscript and acquired funding. All authors discussed the content.

## Conflict of Interest

The authors declare that the research was conducted in the absence of any commercial or financial relationships that could be construed as a potential conflict of interest.
